# Patterns of Systemic Disease Diagnoses among Medical Professionals in Taiwan: Statistical Analysis and Data Mining

**DOI:** 10.3390/ijerph192114017

**Published:** 2022-10-27

**Authors:** Kai-Jie Ma, Jui-Lien Hung, Ming-Hsien Chou, Jong-Yi Wang

**Affiliations:** 1 Department of Public Health, China Medical University, Taichung 406040, Taiwan; 2 Department of Health Services Administration, China Medical University, Taichung 406040, Taiwan; 3 Department of Physical Medicine and Rehabilitation, Taichung Armed Forces General Hospital, Taichung 411228, Taiwan; 4 Department of Physical Medicine and Rehabilitation, Tri-Service General Hospital, Taipei 114202, Taiwan; 5 Section of Physical Medicine and Rehabilitation, School of Medicine, National Defense Medical Center, Taipei 114201, Taiwan

**Keywords:** medical professional, risk of disease, systemic disease, diagnosis sequence, data mining

## Abstract

Introduction: Although high-risk work environments and heavy workload expose medical professionals to long-term risks of disease, no comprehensive analysis has been conducted on the corresponding risks of diseases to each type of medical professionals. This study pre-analyzed the risks of medical professionals in developing various systemic diseases in Taiwan to provide a comprehensive examination of the differences between each type of systemic disease. Methods: From the secondary databases of 2002–2013, 15,407 medical professionals were selected for analysis. A chi-squared test and logistic regression were performed to identify the relationship between types of medical professionals and systemic diseases. The life trajectories of diagnosis sequence of the medical professionals were illustrated accordingly. Results: The physicians were the most vulnerable to infectious, parasitic, and digestive diseases. This was possibly associated with their work characteristics and occupational risks. Conclusion: According to the life trajectories, all types of the medical professionals exhibited a similar trend in the orders of risks to each type of systemic disease, which suggests that their work environment exposes them to real risks of health hazard.

## 1. Introduction

The Ministry of Health and Welfare of Taiwan published the top ten causes of death for 2020. In addition to transportation accidents, the causes of death were neoplasms; endocrine diseases, nutritional diseases, metabolic and immune diseases; digestive diseases; diseases of the circulatory system; diseases of the respiratory system, and diseases of the genitourinary system [1], classified according to the International Classification of Diseases, Ninth Revision (ICD-9). These causes of death have been in the top ten causes of death in Taiwan for more than ten years. According to the World Health Organization (WHO), the top three causes of death worldwide for 2019 were diseases of the circulatory system, diseases of the respiratory system, and certain conditions originating in the perinatal period [2]. The top two causes of death worldwide are similar to those in Taiwan. Furthermore, the WHO has indicated that heart diseases (diseases of the circulatory system) have been a leading cause of death worldwide for the past 20 years. These diseases impose a heavy burden on national insurance and therefore need to be taken seriously, but unfortunately, we do not have any database of death-related cases to explore mortality or survival.

Multiple studies have indicated that medical workers are susceptible to infectious and parasitic diseases [3,4]; neoplasms [5,6]; endocrine diseases, nutritional diseases, metabolic and immune diseases [7,8]; psychosis [9,10]; neurological disorders and eye and adnexa diseases [11,12]; diseases of the circulatory system [6,12]; diseases of the respiratory system [13,14]; digestive diseases [15,16]; diseases of the genitourinary system [15]; diseases of the skin and subcutaneous tissue [17,18], and diseases of the musculoskeletal system and connective tissue [19,20,21]. Many risk factors have been associated with medical workers to develop systemic diseases, such as occupation [6,22], gender [23], age [24], work experience, Charlson comorbidity index [16], payroll bracket, medical institution class [25], medical institution ownership, division of the National Health Insurance Administration, and degree of urbanization [26]. The International Labour Organization (ILO) has reported a high correlation between working environments and occupational diseases [27]. Medical workers are exposed to high-risk working environments for long periods; hence, they are more susceptible to occupational diseases. The ILO has stated that, for a disease to be defined as occupational, a causal relationship must be proven to exist; however, verifying the causal relationship between medical occupations and systemic diseases is difficult. Therefore, whether the aforementioned systemic diseases are definable as occupational disease remains debatable. However, the investigations performed in this study can serve as a reference for relevant institutions seeking to implement precautionary measures in the future.

Although it is generally known that medical providers are in occupations subject to higher risk of contracting disease, there are few effective protections. Furthermore, past studies on the diseases of medical providers have predominantly focused on specific diseases and the environmental risk factors that caused such diseases; they have rarely provided comprehensive analyses of the types of systemic diseases medical workers may have. Therefore, we designed a complete analysis of diseases contracted by medical providers, and chose not to focus on specific diseases. We classified multiple diseases with the same characteristics into one kind of systemic diseases. We expect to be able to predict which medical providers will develop certain kinds of systemic diseases and want to further understand which factors correlate with systemic diseases. This study hypothesized that medical providers will suffer from multiple occupational systemic diseases. We also expect that the results of this study can provide institutions developing national health policies with insights into future formulation of preventive measures to reduce occupational risks for medical workers.

## 2. Methods

### 2.1. Data Sources

This retrospective study used a secondary database. The study period was from 2002 to 2013, and the data were collected from 1 million random records provided by the National Health Insurance Research Database (NHIRD). Established in 1995, NHIRD is the most complete electronic health record in Taiwan. The NHIRD contains demographic variables, outpatient and inpatient information, prescriptions, diagnosis information, medical personnel information, and other detailed clinical information [28]. Our data sources were five types of registration files, Registry for beneficiaries (ID), Registry for contracted medical facilities (HOSB), Registry for medical personnel (PER), Ambulatory care expenditures by visits (CD), and Inpatient expenditures by admissions (DD). Our study was approved by the Research Ethics Committee of China Medical University Hospital, Taiwan. To protect the patients’ privacy, all personal identification numbers were encrypted by the National Health Research Institutes before the data were released. The Taiwan National Health Research Institutes encrypts patients’ personal information to protect privacy and provides researchers with anonymous scrambled identification numbers associated with relevant disease information. Therefore, a patient informed consent is not required for authorized researchers to access this research database.

### 2.2. Inclusion and Exclusion Criteria

Our study included 77,035 people with systemic diseases, and systemic diseases defined according to the ICD-9 standard from the 2002 to 2013 NHIRD database. Later, we excluded 228 people whose incomplete data or lost date of diagnosis. Then, we divided the study sample into two groups: non-medical professionals and medical professionals (N = 15,179). In a second stage, we divided the medical professionals into three groups according to occupation, e.g., physician, nursing personnel, and other medical personnel. Other medical personnel included pharmacists, midwives, medical technologists, dental technicians, physical therapists, radiographers, counseling psychologists, dietitians, and social workers (Figure S1).

### 2.3. ICD-9 of Systemic Diseases

Our research objectives were to explore the relationship between medical providers and diseases. The authors try to cover all diseases; however, there are currently more than a hundred thousand ICD-9 codes. Therefore, we referred to the top 10 causes of death published in Taiwan, and identified 11 disease categories as follows: infectious and parasitic diseases; neoplasms; endocrine, nutritional, metabolic, and immune diseases; psychosis; neurological disorders and eye and adnexa diseases; diseases of the circulatory system; diseases of the respiratory system; digestive diseases; diseases of the genitourinary system; diseases of the skin and subcutaneous tissue; and diseases of the musculoskeletal system and connective tissue. The codes are presented in Table 1 below.

### 2.4. Charlson Comorbidities Index

Our research used the Charlson Comorbidity Index (CCI) developed by Charlson in 1984, to evaluate the mortality risk and burden of disease, address the confounding influence of comorbidities, and predict outcomes. We followed the method proposed by Charlson; the CCI consists of 17 comorbidities, weighted from 1 to 6 according to mortality risk and disease severity, and then summed scores to form the total CCI score [29]. However, subjects rarely displayed high CCI scores in our research, so we divided the CCI categories into three groups as follows: 0 points, 1 point, and more than 2 points.

### 2.5. Statistical Analysis

First, we used adjusted logistic regression and a 95% confidence interval to compare the risk of developing systemic disease between workers and non-medical professionals and medical professionals. Second, we further analyzed the association of the three groups among medical professionals with developing systemic diseases. We used descriptive statistics, namely frequencies and percentages, to understand the sample distribution with respect to each variable. The chi-squared test of independence was used to test the correlation between occupations and the 11 systemic diseases. Collinearity diagnostics were performed to verify whether the independent variables had high correlations with each other; the diagnostic results did not reveal any collinearity. Adjusted logistic regression and a 95% confidence interval were used to investigate the risk of developing systemic diseases in relation to each independent variable. Finally, we designed the first day of work for medical professional as a baseline, and we calculated the time from the baseline to the diagnosis-date for each individual, and calculated separately for each systemic disease. We used the average developing years of each systemic disease to plot a life trajectory, and to explore how long after working do medical providers develop systemic diseases. These analyses were conducted using SPSS 22 software.

## 3. Results

### 3.1. Non-Medical Professionals and Medical Professionals

We divided the study samples into two groups: medical professionals and non-medical professionals. The medical professional group has 15,179 (19.76%) study samples and the non-medical professional group has 61,628 (80.24%) study samples. All variable diagnostic results did not reveal any collinearity after collinearity diagnostics. After adjusting for logistic regression analysis, medical professionals displayed a 1.160 times higher risk than non-medical professionals. We further analyzed the risk of each systemic disease between those two groups and found that the medical professional group displayed a higher risk of infectious and parasitic diseases; neoplasms; digestive diseases, and diseases of the skin and subcutaneous tissue, and conversely, diseases of the respiratory system and diseases of the musculoskeletal system and connective tissue displayed a higher risk for the non-medical professionals (Table S1).

### 3.2. Participant Characteristics

The study sample comprised the data of 15,179 Taiwanese medical workers. Table 2 illustrates the sample distribution with respect to each variable; 24.28% were male and 75.72% were female. The medical workers were classified as physicians (12.83%), nursing personnel (59.13%), or other medical personnel (28.04%). The average age of the medical workers was 39.67 ± 12.87 years. A higher proportion of the workers had a low Charlson comorbidity index score; had a small amount of insured salary; worked in clinics, private hospitals, or private institutions; belonged to the Taipei National Health Insurance Regional Division; lived in highly urbanized areas; and had a small amount of work experience.

### 3.3. Association between Person Type of Medical and Category Disease

The results, presented in Table 3, revealed a correlation of occupation with 10 of the 11 systemic disease categories, namely infectious and parasitic diseases; endocrine diseases, nutritional, metabolic, and immune diseases; psychosis; neurological disorders and eye and adnexa diseases; diseases of the circulatory system; diseases of the respiratory system; digestive diseases; diseases of the genitourinary system; diseases of the skin and subcutaneous tissue; and diseases of the musculoskeletal system and connective tissue. When the demographic and regional variables were controlled, physicians were 1.376 times at higher risk than other medical personnel of developing infectious and parasitic diseases (*p* = 0.020, odds ratio (OR) = 1.376) and 1.402 times higher risk than other medical personnel of contracting digestive diseases (*p* < 0.001, OR = 1.402). As indicated in Table 4, male medical providers showed 1.426 times (*p* = 0.009, OR = 1.426) higher risk of developing infectious and parasitic diseases than female providers, and medical providers with CCI scores of 0 and 1 were at 2.782 (*p* = 0.044, OR = 2.782) and 2.909 times (*p* = 0.038, OR = 2.909) higher risk of developing risk of infectious and parasitic diseases than medical providers with CCI scores of more than 2 points. Medical workers who worked in medical centers displayed 1.645 times (*p* = 0.016, OR = 1.646) higher risk of developing infectious and parasitic diseases than those working in district hospitals. In addition, medical workers living in Taipei (*p* = 0.003, OR = 1.578), Northern (*p* = <0.001, OR = 1.897), Central (*p* = <0.001, OR = 2.129), Southern (*p* = <0.001, OR = 2.033) and Eastern Divisions (*p* = 0.037, OR = 1.952) were all at higher risk of developing infectious and parasitic diseases than medical workers who lived in the Kaoping Division. Medical workers living in moderately urbanized, emerging, and agricultural areas were at 2.131 to 2.510 times higher risk of developing infectious and parasitic diseases than those living in remote areas. In addition, in Table 5, male medical providers (*p* = 0.011, OR = 1.171) were at 1.171 times higher risk of contracting digestive diseases than female medical providers, and medical providers who worked in regional hospitals (*p* = 0.021, OR = 1.332) and clinics (*p* < 0.001, OR = 1.713) displayed 1.332 and 1.713 times higher risk of developing digestive diseases than those who worked in district hospitals. Medical providers working in private hospitals or institutions (*p* < 0.001, OR = 1.809) displayed 1.809 times higher prevalence of digestive diseases than those working in public hospitals or institutions, and medical providers who belonged to the Taipei (*p* < 0.001, OR = 1.341) and the Northern Division (*p* = 0.004, OR = 1.240) displayed 1.341 and 1.240 times higher prevalence of digestive diseases than those belonging to the Kaoping Division.

### 3.4. Life Trajectory

**Figure 1:**
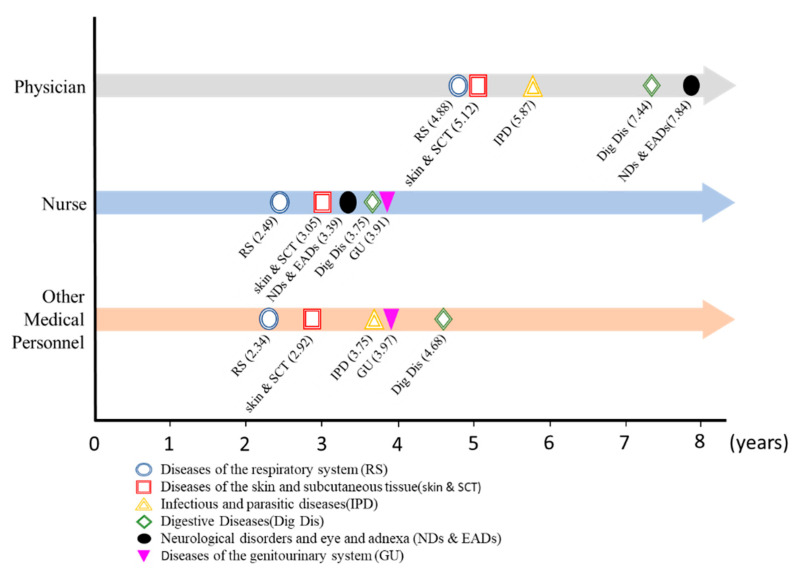
Time between beginning of career and development of systemic diseases among medical professionals in Taiwan.

The results for the three occupation categories are listed in Figure 1. The top five systemic diseases of diagnosis sequence in each category were identified. On average, the physician group developed diseases of the respiratory system most quickly in time sequence (within 4.88 years), followed by diseases of the skin and subcutaneous tissue; infectious and parasitic diseases; digestive diseases, neurological disorders, and eye and adnexa diseases. The top five diseases for all three occupation categories included diseases of the respiratory system, diseases of the skin and subcutaneous tissue, and digestive diseases. In addition, for all three categories of occupation, diseases of the respiratory system and diseases of the skin and subcutaneous tissue were the first and second fastest diseases to develop, respectively. This result warrants further verification through research in the future. We also discovered that the average period before physicians started developing systemic diseases was longer than it was for the other two groups. This could be attributed to the health awareness and behaviors of physicians.

## 4. Discussion

In this study, first, we examined 11 systemic diseases between medical workers and non-medical workers. We found that medical workers have a higher risk (*p* < 0.001, OR = 1.160) to be diagnosed with the systemic disease than non-medical workers after adjusting the control factors. Next, we used the ICD-9 to categorize 11 systemic diseases that medical workers were susceptible to and investigated the difference between the occurrence of systemic diseases in physicians, nursing personnel, and other medical personnel. The results revealed that nursing personnel had the highest prevalence of systemic diseases (59.13%). In addition, 10 systemic diseases had significant relationships with occupation; only neoplasms did not. We further controlled related influencing factors and revealed significant relationships of infectious and parasitic diseases and digestive diseases with occupation. The risk of developing such diseases in the physician group (OR = 1.376 and 1.402, respectively) was higher than that in the other two groups. These results are consistent with the results of a previous research study, which included 90 nursing students and 110 medical residents in hospitals in India; the research study found that medical residents were at two-fold greater risk of incident latent tuberculosis infection than nursing students (Relative Risk, 2.16; 95% CI, 1.05–4.42) [30]. In addition, our results for infectious and parasitic diseases may be explained by the results of a past review research, which indicated that health care workers are more likely to be exposed to infectious and parasitic diseases, but this study does not specify whether the workers are physicians or nurses [31]. One previous research study exploring the incidence rate between physicians and the general population found that physicians had higher incidence rates of endocrine, nutritional, metabolic, and immune diseases [30], but we did not find this characteristic in our research. Finally, this study investigated the life trajectory of medical workers who developed the 11 systemic diseases after they started working in the medical industry and revealed that the median time before physicians developed infectious and parasitic diseases or digestive diseases was longer than that for nursing or other medical personnel.

### 4.1. Non-Medical Professionals and Medical Professionals

Our study used logistic regression and adjusting other control variables. We found that medical professionals displayed higher risk than non-medical professionals to be diagnosed with systemic diseases. One past study found the increased time spent in healthcare by medical professionals as an important risk factor for latent TB infection [32], which is consistent with our results. Another study about asthma displayed that male healthcare workers were at higher risk to suffer from asthma [33], but we did not find this result in our study. In addition, our research displayed that medical professionals have a higher risk of infectious and parasitic diseases; neoplasms; digestive diseases, and diseases of the skin and subcutaneous tissue; so, we further subdivided medical professionals into three groups: physician, nursing personnel, and other medical personnel.

### 4.2. Factors Correlated to the Development of Systemic Diseases in Medical Workers

We used the chi-squared test of independence and discovered that the 10 systemic diseases (excluding only neoplasms) significantly differed with occupation. Other studies have identified a correlation of systemic diseases with occupation, gender, age, Charlson comorbidity index, amount of insured salary, medical institution class, medical institution ownership, division of National Health Insurance Administration, degree of urbanization, and work experience. Therefore, this study included these factors and adjusted them to obtain the following results. Physicians had a high risk of infectious and parasitic diseases or digestive diseases, with ORs of 1.376 and 1.402, respectively. This indicated that physicians were susceptible to digestive diseases, which supports the results of past studies [34,35].

### 4.3. Risk of Medical Workers Developing Systemic Diseases

An adjusted logistic regression analysis revealed that infectious and parasitic diseases and digestive diseases were significantly correlated with occupation. We believe that the possible reason physicians are susceptible to infectious and parasitic diseases (e.g., Helminthiasis, Tuberculosis, Hepatitis B/C) may be related to the characteristics of their work. A plausible reason is perhaps that most physicians in Taiwan will participate in one or more clinical research projects, e.g., microbiological experiments and drug experiments that increase the chance of exposure to infectious sources [36,37]. Past research has shown that laboratory workers are more susceptible to hepatitis B infection [38]. A second possible reason, physicians in Taiwan also have a higher rate of syringe prick injury than other medical providers except for nurses [39]. A few years ago, some experts proposed that transmission of infectious microorganisms poses a threat not just to healthcare workers in direct contact with patients, but can also be spread via contaminated hands, apparel/uniforms, patient care items (e.g., IV poles, privacy curtains, blood pressure cuffs) or environmental surfaces [40]. Both may cause the spread of infectious diseases; in addition, failure to use appropriate PPE will increase the risk of exposure to splashes and splatters at work [40]. As a third possible reason, we thought that the working hours of physicians may also play a role. The findings reveal that the average total work hours per a week of an attending physician in Taiwan is around 69.1 h [41], physicians in US work about 50 h per week [42,43], nurses in Taiwan work about 50 to 60 h per a week [44,45]; physicians in Taiwan work longer than physicians in the US or nurses in Taiwan. Long-time face-to-face patient meetings indirectly increase the prevalence of infectious and parasitic diseases. In addition, there is a high incidence of burnout among Taiwan physicians, which will cause negligence in work [46].

The observation that physicians are at a higher risk of developing digestive diseases (e.g. Peptic Ulcer; Gastritis) is possibly due to their long working hours [34]. In Taiwanese hospitals, physicians working in outpatient clinics generally have numerous patients, and they require extensive time to provide all of these patients with diagnoses. Therefore, physicians often use their lunch breaks or work overtime to treat patients, which prevents them from keeping life routines and this may increase their risk of developing digestive diseases. This study also revealed that medical workers working in clinics in the Taipei or Northern Divisions of the National Health Insurance Administration had the highest risk of developing digestive diseases. This could be due to the reimbursement system of the National Health Insurance of Taiwan. According to data published by the Department of Statistics of the Ministry of Health and Welfare, more than 30% of all clinics in Taiwan are dental clinics [1]. Dental clinics have more self-payment service items than other types of clinics, and they do not have a limit on the number of patients they can receive. Because treating more patients leads to a higher income, physicians working in such clinics work overtime, thereby disrupting their life routines and maybe increasing their risk of developing digestive diseases.

### 4.4. Diagnosis Sequence of Systemic Diseases

In our study, we design the worker’s career start date as the baseline for the life trajectory, and listed the top five systemic diseases that medical workers developed most quickly after they began working in medical institutions and compared the results between the three categories of medical personnel. Both the types and order (regarding the time sequence of development) of diseases were similar between the three categories. This may indicate that the reason for medical workers to develop systemic diseases was not due to personal factors or habits. Rather, the diseases may have resulted from a similar working environment or other common factors. Although the results of Table 2 indicate that only two systemic diseases were significant with the occupations in our research, a similar diagnosis sequence observed in Figure 1 is still exciting. The diagnosis sequences in three occupations are coincidentally similar, this diagnosis sequence result may indirectly indicate that the systemic diseases in Figure 1 present significantly with certain occupations; and the authors may be limited by the database or undiscovered influencing factors. Future studies may further investigate this topic. This study also revealed that the average period before physicians developed systemic diseases was longer than that for the other two groups. This may be because physicians have more knowledge related to medicine and diseases [47].

## 5. Conclusions

Occupation as a physician was associated with infectious, parasitic, and digestive diseases. Particularly, sexes, comorbidity, types of contracts with medical institutions, insurance branches, and urbanization levels were factors presenting significant correlations with the medical professionals’ risks of disease. Furthermore, according to the life trajectories plotted in this study, a consistency was observed in the 3 groups in terms of the highest annual average occurrences, with RS occurring the most frequently, followed by skin and SCT, and Dig Dis. Accordingly, career risks are critical to medical professionals’ risks of disease. Health authorities must endeavor to promote preventive medicine education to improve the health and well-being of medical professionals.

### 5.1. Strengths

This study’s review of data, which was representative of medical workers across Taiwan, revealed potential factors correlated to systemic disease development in medical workers, such as their gender, age, occupation, and Charlson comorbidity index. The medical workers’ risk of developing diseases was analyzed to facilitate development of preventive measures for high-risk groups. This study can serve as a reference for medical institutions and government agencies planning preventive intervention measures. In addition, our research identified types of systemic diseases shared by different categories of medical workers. The results can serve as a reference for medical institutions and government agencies in planning disease prevention interventions.

### 5.2. Limitations

Our research had four limitations. First, our database used in this study did not include the average working time of medical personnel. The average working time may be a crucial factor leading medical workers to develop diseases. In addition, there are no discipline data in the NHIRD database, so we cannot classify the disciplines to which the study samples belong; secondly, other crucial correlation factors that may have contributed to medical workers to develop the included systemic diseases could not be obtained from the National Health Insurance Research Database. Such factors include family history, educational attainment, marital status, religion, work stress, and personality traits. Third, when we identified this research project, the 2002–2013 NHIRD was the most current source that allowed us to address our key populations. Although the data are not as current as we would like, but it is a database with 12 years of data. Our results can establish a baseline for future research. Fourth, our definition of control participants was based on systemic diseases ICD-9 codes and did not include other potential indicators of diseases. Fifth, after the COVID-19 pandemic, the risk of infection among medical personnel has increased significantly, which may reverse the results of the past, and it may be interesting to explore the trend of morbidity rates before and after the COVID-19 pandemic.

## Figures and Tables

**Table 1:** ICD-9-CM codes to systemic diseases.

	ICD-9	Disease
1	001.0–139.8	Infectious and parasitic disease
2	140.0–239.9	Neoplasms
3	240.0–279.9	Endocrine, nutritional, metabolic, and immune Diseases
4	290.0–319	Psychosis
5	320.0–389.9	Neurological disorders and eye and adnexa Diseases
6	390–459.9	Diseases of the circulatory system
7	460–519.9	Diseases of the respiratory system
8	520.0–579.9	Digestive diseases
9	580.0–629.0	Diseases of the genitourinary system
10	680.0–709.9	Diseases of the skin and subcutaneous tissue
11	710.0–739.9	Diseases of the musculoskeletal system and connective tissue

**Table 2:** Characteristics of the samples studied (N = 15,179).

Variables	N	%
Gender		
Male	3686	24.28
Female	11,493	75.72
Age		
20–29	3462	22.81
30–39	5358	35.30
40–49	3146	20.73
50–59	1993	13.13
older than 60	1220	8.04
Charlson comorbidity index (CCI)		
0	12,030	79.25
1	2848	18.76
2 points or more	301	1.98
Payroll bracket (New Taiwan Dollar, NTD)		
less than 22,800	11,221	73.92
22,801–36,300	1459	9.61
36,301–45,800	1025	6.75
more than 45,801	1474	9.71
Medical institution class		
Medical center	1683	11.09
Regional hospital	1242	8.18
District hospital	1307	8.61
Clinic	10,923	71.96
Medical institution ownership		
Public hospital or institution	1282	8.45
Private hospital or institution	12,196	80.35
Medical foundation	1522	10.03
Medical corporation	135	0.89
National Health Insurance Administration division		
Taipei Division	5102	33.61
Northern Division	1709	11.26
Central Division	2909	19.16
Southern Division	2313	15.24
Kaoping Division	2841	18.72
Eastern Division	305	2.01
Degree of urbanization		
Highly urbanized area	4994	32.90
Moderately urbanized area	4892	32.23
Emerging area	1871	12.33
General area	2033	13.39
Aging area	362	2.38
Agricultural area	534	3.52
Remote area	493	3.25
Occupation		
Physician	1947	12.83
Nursing personnel	8976	59.13
Other medical personnel	4256	28.04
Work experience		
<5 years	5301	34.92
6–10 years	4011	26.42
11–15 years	3417	22.51
≥16 years	2450	16.14

**Table 3:** Association of different medical provider in Taiwan with systemic diseases (N = 15,179).

	%	*p*	aOR	*p*
Infectious and parasitic diseases				
Physician	6.0	<0.001	1.376	0.020 *
Nursing personnel	3.3		1.007	0.954
Other medical personnel (Ref.)	3.7		-	
Neoplasms				
Physician	2.0	0.170	1.285	0.285
Nursing personnel	1.6		1.088	0.645
Other medical personnel (Ref.)	1.4		-	
Endocrine, nutritional, metabolic and Immune diseases				
Physician	3.5	<0.001	1.060	0.753
Nursing personnel	1.4		0.960	0.825
Other medical personnel (Ref.)	2.0		-	
Psychosis				
Physician	1.8	0.001	0.780	0.268
Nursing personnel	1.1		0.737	0.110
Other medical personnel (Ref.)	1.8		-	
Neurological disorders and eye and adnexa				
Physician	9.6	<0.001	0.988	0.909
Nursing personnel	7.3		0.873	0.095
Other medical personnel (Ref.)	9.0		-	
Diseases of the circulatory system				
Physician	4.3	<0.001	0.870	0.414
Nursing personnel	1.0		0.871	0.485
Other medical personnel (Ref.)	2.5		-	
Diseases of the respiratory system				
Physician	20.9	<0.001	0.926	0.285
Nursing personnel	25.7		1.034	0.524
Other medical personnel (Ref.)	24.1		-	
Digestive Diseases				
Physician	31.6	<0.001	1.402	<0.001 **
Nursing personnel	22.6		0.928	0.157
Other medical personnel (Ref.)	25.6		-	
Diseases of the genitourinary system				
Physician	3.8	<0.001	0.995	0.974
Nursing personnel	9.7		1.091	0.280
Other medical personnel (Ref.)	6.0		-	
Diseases of the skin and subcutaneous tissue				
Physician	8.8	<0.001	1.073	0.498
Nursing personnel	12.0		1.027	0.704
Other medical personnel (Ref.)	10.1		-	
Diseases of the musculoskeletal system and connective tissue				
Physician	7.6	<0.001	0.923	0.486
Nursing personnel	5.2		0.867	0.133
Other medical personnel (Ref.)	6.9		-	

* *p* < 0.05, ** *p* < 0.01.

**Table 4:** Multivariable Analysis of Associations Between medical providers and infectious and parasitic disease (N = 15,179).

Variables	aOR	95% CI			*p*
Occupation					
Physician	1.376	1.051	-	1.802	0.020 *
Nursing personnel	1.007	0.790	-	1.285	0.954
Other medical personnel (Ref.)	-			-	-
Gender					
Male	1.426	1.092	-	1.863	0.009 *
Female (Ref.)	-			-	-
Age					
20–29	1.337	0.815	-	2.191	0.250
30–39	1.270	0.834	-	1.933	0.265
40–49	1.310	0.903	-	1.901	0.154
50–59	0.983	0.666	-	1.451	0.932
older than 60 (Ref.)	-			-	-
Charlson comorbidity index (CCI)					
0	2.782	1.026	-	7.543	0.044 *
1	2.909	1.063	-	7.964	0.038 *
2 points or more (Ref.)	-			-	-
Payroll bracket (New Taiwan Dollar, NTD)					
less than 22,800	1.092	0.775	-	1.541	0.614
22,801–36,300	0.792	0.526	-	1.193	0.265
36,301–45,800	0.965	0.624	-	1.494	0.874
more than 45,801 (Ref.)	-			-	-
Medical institution class					
Medical center	1.646	1.098	-	2.466	0.016 *
Regional hospital	1.352	0.846	-	2.161	0.207
District hospital (Ref.)	-			-	-
Clinic	0.878	0.635	-	1.213	0.430
Medical institution ownership					
Public hospital or institution (Ref.)	-			-	-
Private hospital or institution	1.091	0.743	-	1.602	0.656
Medical foundation	0.857	0.597	-	1.229	0.401
Medical corporation	<0.001	<0.001->999.999			0.996
National Health Insurance Administration division					
Taipei Division	1.578	1.171	-	2.127	0.003 *
Northern Division	1.897	1.332	-	2.701	<0.001 *
Central Division	2.129	1.563	-	2.900	<0.001 *
Southern Division	2.033	1.462	-	2.826	<0.001 *
Kaoping Division (Ref.)	-			-	-
Eastern Division	1.952	1.040	-	3.663	0.037 *
Degree of urbanization					
Highly urbanized area	2.333	1.125	-	4.839	0.023 *
Moderately urbanized area	2.510	1.221	-	5.158	0.012 *
Emerging area	2.131	1.010	-	4.497	0.047 *
General area	2.098	1.001	-	4.394	0.050
Aging area	1.709	0.680	-	4.292	0.254
Agricultural area	2.355	1.024	-	5.412	0.044 *
Remote area (Ref.)	-			-	-
Work experience					
<5 years	0.792	0.548	-	1.143	0.212
6–10 years	0.870	0.628	-	1.206	0.404
11–15 years	0.967	0.720	-	1.298	0.822
≥16 years (Ref.)	-			-	-

* *p* < 0.05.

**Table 5:** Multivariable analysis of associations between medical providers and digestive diseases (N = 15,179).

Variable	aOR	95% CI			*p*
Occupation					
Physician	1.402	1.230	-	1.598	<0.001 *
Nursing personnel	0.928	0.836	-	1.029	0.157
Other medical personnel (Ref.)	-			-	-
Gender					
Male	1.171	1.037	-	1.323	0.011 *
Female (Ref.)	-			-	-
Age					
20–29	0.849	0.686	-	1.050	0.130
30–39	0.888	0.739	-	1.067	0.204
40–49	0.986	0.837	-	1.162	0.867
50–59	0.993	0.840	-	1.173	0.932
older than 60 (Ref.)	-			-	-
Charlson comorbidity index (CCI)					
0	0.931	0.711	-	1.220	0.606
1	1.137	0.862	-	1.500	0.364
2 points or more (Ref.)	-			-	-
Payroll bracket (New Taiwan Dollar, NTD)					
less than 22,800	1.109	0.948	-	1.297	0.195
22,801–36,300	0.991	0.831	-	1.181	0.916
36,301–45,800	1.188	0.984	-	1.434	0.074
more than 45,801 (Ref.)	-			-	-
Medical institution class					
Medical center	1.116	0.899	-	1.386	0.318
Regional hospital	1.332	1.045	-	1.699	0.021 *
District hospital (Ref.)	-			-	-
Clinic	1.713	1.461	-	2.010	<0.001 *
Medical institution ownership					
Public hospital or institution (Ref.)	-			-	-
Private hospital or institution	1.809	1.465	-	2.234	<0.001 *
Medical foundation	1.154	0.934	-	1.428	0.185
Medical corporation	1.022	0.610	-	1.712	0.936
National Health Insurance Administration division					
Taipei Division	1.341	1.194	-	1.506	<0.001 *
Northern Division	1.240	1.072	-	1.435	0.004 *
Central Division	1.076	0.947	-	1.222	0.263
Southern Division	0.956	0.831	-	1.099	0.524
Kaoping Division (Ref.)	-			-	-
Eastern Division	1.283	0.965	-	1.705	0.086
Degree of urbanization					
Highly urbanized area	0.844	0.674	-	1.057	0.140
Moderately urbanized area	0.804	0.645	-	1.003	0.053
Emerging area	0.796	0.628	-	1.009	0.059
General area	0.908	0.720	-	1.145	0.413
Aging area	0.848	0.608	-	1.183	0.332
Agricultural area	0.934	0.699	-	1.248	0.644
Remote area (Ref.)	-			-	-
Work experience					
<5 years	1.023	0.875	-	1.196	0.775
6–10 years	0.981	0.851	-	1.130	0.791
11–15 years	0.943	0.827	-	1.075	0.379
≥16 years (Ref.)	-			-	-

* *p* < 0.05.

## Data Availability

This study used national databases obtained from the Health and Welfare Data Science Center (HWDC), Ministry of Health and Welfare in Taiwan. All data obtained were anonymized and deidentified by the HWDC. The data used in this study must be accessed and analyzed in the HWDC under its regulation after filling out an application and thus cannot be shared. However, such data can be accessed from the authors upon reasonable request and with permission from HWDC (https://dep.mohw.gov.tw/DOS/cp-2516-59203-113.html, accessed on 1 January 2022). Data are also available from the China medical University & Hospital Research Ethics Committee (http://61.66.117.10/2007/IRB/index.html, accessed on 1 January 2022) for researchers who satisfy the criteria for accessing confidential data.

## Supplementary Materials

The following supporting information can be downloaded at: https://www.mdpi.com/article/10.3390/ijerph192114017/s1, Figure S1. Flow chart. Table S1. Association between medical professionals and non-medical professionals in Taiwan with systemic diseases (N = 75,981).
