# Supplementary material for: Patterns of Systemic Disease Diagnoses among Medical Professionals in Taiwan: Statistical Analysis and Data Mining
Source: Int J Environ Res Public Health. 2022 Oct 27;19(21):14017. doi: 10.3390/ijerph192114017 (PMC9657375; doi:10.3390/ijerph192114017)
Supplement: Supplementary file 1 [file ijerph-19-14017-s001.zip › ijerph-1907420-supplementary.pdf]

Supporting Information

Figure S1
Flow chart

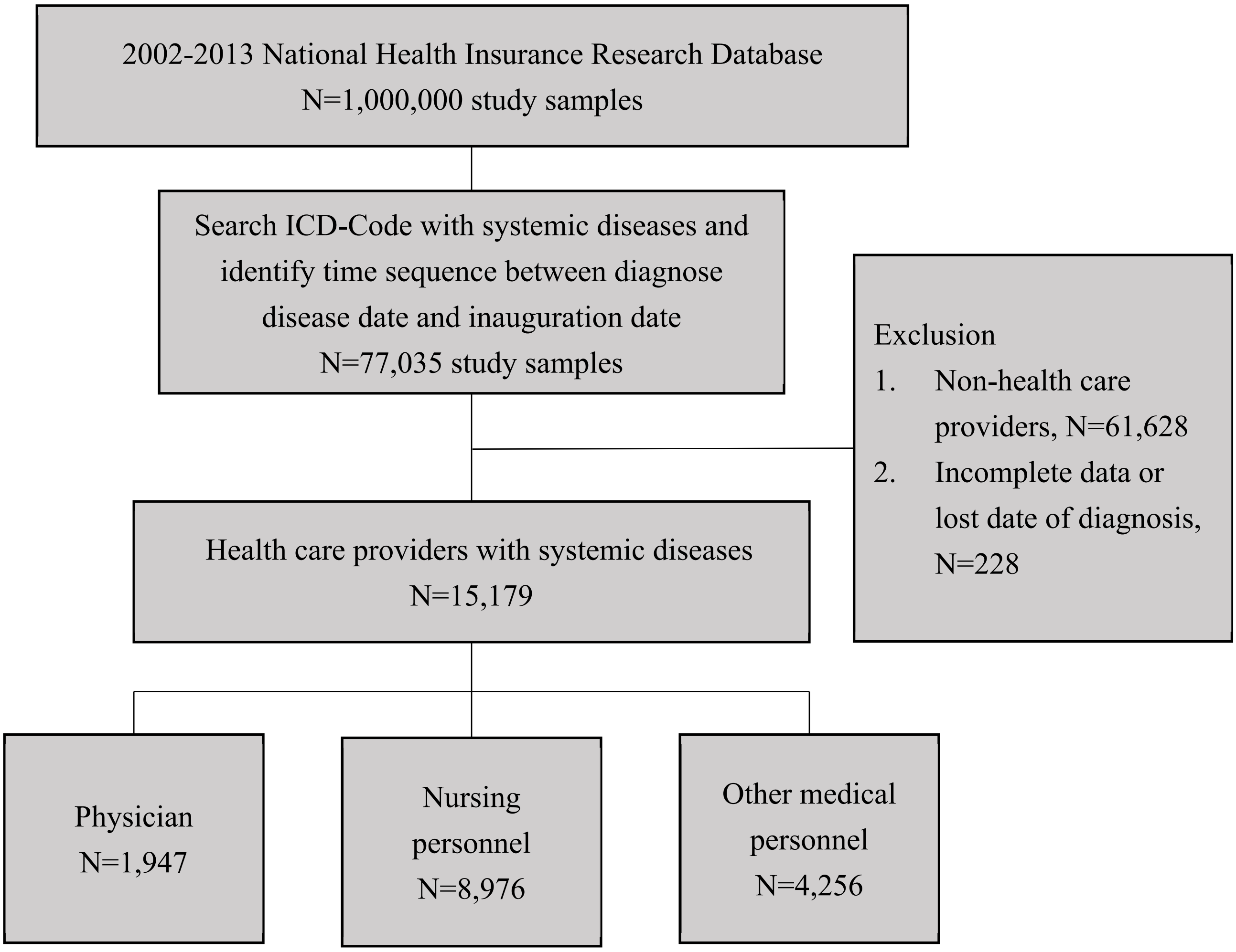

Table S1. Association between medical professionals and non-medical professionals in Taiwan with systemic diseases(N=75,981)

	Adjusted Odd Risk	95% CI			*p* 值
**Systemic diseases**					
non-medical professionals (Ref.)	-			-	-
medical professionals	1.160	1.101	-	1.223	<0.001*
**Infectious and parasitic diseases**					
non-medical professionals (Ref.)	-			-	-
medical professionals	1.196	1.083	-	1.321	<0.001*
**Neoplasms**					
non-medical professionals (Ref.)	-			-	-
medical professionals	1.173	1.003	-	1.372	0.045*
**Endocrine, nutritional, metabolic and Immune diseases**					
non-medical professionals (Ref.)	-			-	-
medical professionals	1.064	0.919	-	1.233	0.407
**Psychosis**					
non-medical professionals (Ref.)	-			-	-
medical professionals	0.942	0.801	-	1.107	0.469
**Neurological disorders and eye and adnexa**					
non-medical professionals (Ref.)	-			-	-
medical professionals	1.005	0.939	-	1.076	0.886
**Diseases of the circulatory system**					
non-medical professionals (Ref.)	-			-	-
medical professionals	1.042	0.900	-	1.205	0.584
**Diseases of the respiratory system**					
non-medical professionals (Ref.)	-			-	-
medical professionals	0.874	0.838	-	0.912	<0.001*
**Digestive Diseases**					
non-medical professionals (Ref.)	-			-	-
medical professionals	1.254	1.201	-	1.310	<0.001*
**Diseases of the genitourinary system**					
non-medical professionals (Ref.)	-			-	-
medical professionals	1.014	0.947	-	1.087	0.684
**Diseases of the skin and subcutaneous tissue**					
non-medical professionals (Ref.)	-			-	-
medical professionals	1.099	1.036	-	1.166	0.002*

**Diseases of the musculoskeletal system and connective tissue**					
non-medical professionals (Ref.)	-			-	-
medical professionals	0.866	0.801	-	0.937	<0.001*

* $p < 0.05$, ** $p < 0.001$.
